# JAK/STAT-Activating Genomic Alterations Are a Hallmark of T-PLL

**DOI:** 10.3390/cancers11121833

**Published:** 2019-11-21

**Authors:** Linus Wahnschaffe, Till Braun, Sanna Timonen, Anil K. Giri, Alexandra Schrader, Prerana Wagle, Henrikki Almusa, Patricia Johansson, Dorine Bellanger, Cristina López, Claudia Haferlach, Marc-Henri Stern, Jan Dürig, Reiner Siebert, Satu Mustjoki, Tero Aittokallio, Marco Herling

**Affiliations:** 1 Department I of Internal Medicine, Center for Integrated Oncology (CIO), Aachen-Bonn-Cologne-Duesseldorf, University of Cologne (UoC), 50937 Cologne, Germany; linus.wahnschaffe@uk-koeln.de (L.W.); till.braun@uk-koeln.de (T.B.); alexandra.schrader@uk-koeln.de (A.S.); 2 Excellence Cluster for Cellular Stress Response and Aging-Associated Diseases (CECAD), University of Cologne, 50937 Cologne, Germany; prerana.wagle@uk-koeln.de; 3 Center for Molecular Medicine Cologne (CMMC), University of Cologne, 50937 Cologne, Germany; 4 Hematology Research Unit Helsinki, Helsinki University Hospital Comprehensive Cancer Center, FI-00029 Helsinki, Finland; sanna.timonen@helsinki.fi (S.T.); satu.mustjoki@helsinki.fi (S.M.); 5 Translational Immunology Research program and Department of Clinical Chemistry and Hematology, University of Helsinki, FI-00014 Helsinki, Finland; 6 Institute for Molecular Medicine Finland (FIMM), University of Helsinki, FI-00014 Helsinki, Finland; anil.kumar@helsinki.fi (A.K.G.); henrikki.almusa@helsinki.fi (H.A.); tero.aittokallio@helsinki.fi (T.A.); 7 Department of Hematology, University Hospital Essen, University of Duisburg-Essen, 45147 Essen, Germany; patricia.johansson@uk-essen.de (P.J.); jan.duerig@uk-essen.de (J.D.); 8 Institut Curie, Centre de Recherche, 75248 Paris, France; dorine.bellanger@curie.fr (D.B.); marc-henri.stern@curie.fr (M.-H.S.); 9 French National Institute of Health and Medical Research (INSERM) U830, 75248 Paris, France; 10 Institute for Human Genetics, Christian-Albrechts-University Kiel & University Hospital Schleswig Holstein, 24105 Kiel, Germany; cristina.lopez@uni-ulm.de (C.L.); reiner.siebert@uni-ulm.de (R.S.); 11 Institute of Human Genetics, Ulm University, Ulm University Medical Center, D-89081 Ulm, Germany; 12 MLL Munich Leukemia Laboratory, Max-Lebsche-Platz 31, 81377 Munich, Germany; claudia.haferlach@mll.com; 13 German Cancer Consortium (DKTK), 69120 Heidelberg, Germany; 14 Department of Mathematics and Statistics, University of Turku, FI-20014 Turku, Finland

**Keywords:** JAK, STAT, T-PLL, T-cell leukemia, meta-analysis, STAT5B signaling

## Abstract

T-cell prolymphocytic leukemia (T-PLL) is a rare and poor-prognostic mature T-cell leukemia. Recent studies detected genomic aberrations affecting *JAK* and *STAT* genes in T-PLL. Due to the limited number of primary patient samples available, genomic analyses of the JAK/STAT pathway have been performed in rather small cohorts. Therefore, we conducted—via a primary-data based pipeline—a meta-analysis that re-evaluated the genomic landscape of T-PLL. It included all available data sets with sequence information on *JAK* or *STAT* gene loci in 275 T-PLL. We eliminated overlapping cases and determined a cumulative rate of 62.1% of cases with mutated *JAK* or *STAT* genes. Most frequently, *JAK1* (6.3%), *JAK3* (36.4%), and *STAT5B* (18.8%) carried somatic single-nucleotide variants (SNVs), with missense mutations in the SH2 or pseudokinase domains as most prevalent. Importantly, these lesions were predominantly subclonal. We did not detect any strong association between mutations of a *JAK* or *STAT* gene with clinical characteristics. Irrespective of the presence of gain-of-function (GOF) SNVs, basal phosphorylation of STAT5B was elevated in all analyzed T-PLL. Fittingly, a significant proportion of genes encoding for potential negative regulators of STAT5B showed genomic losses (in 71.4% of T-PLL in total, in 68.4% of T-PLL without any *JAK* or *STAT* mutations). They included *DUSP4, CD45, TCPTP, SHP1, SOCS1, SOCS3,* and *HDAC9*. Overall, considering such losses of negative regulators and the GOF mutations in *JAK* and *STAT* genes, a total of 89.8% of T-PLL revealed a genomic aberration potentially explaining enhanced STAT5B activity. In essence, we present a comprehensive meta-analysis on the highly prevalent genomic lesions that affect genes encoding JAK/STAT signaling components. This provides an overview of possible modes of activation of this pathway in a large cohort of T-PLL. In light of new advances in JAK/STAT inhibitor development, we also outline translational contexts for harnessing active JAK/STAT signaling, which has emerged as a ‘secondary’ hallmark of T-PLL.

## 1. Introduction

T-cell prolymphocytic leukemia (T-PLL) is an aggressive malignancy characterized by an expansion of mature T-lymphocytes [1]. Although with an incidence of <2.0/million/year in Western countries infrequently encountered, T-PLL is the most common mature T-cell leukemia [2]. Patients suffering from T-PLL typically present with exponentially rising white blood cell (WBC) counts accompanied by bone marrow (BM) infiltration and splenomegaly, and at lower frequencies by various other manifestations such as effusions or in skin [3]. Due to its chemotherapy-refractory behavior, T-PLL patients have a dismal prognosis with a median overall survival (OS) of <3 years [4,5,6]. Clinicians treating T-PLL face limited therapeutic options, mainly caused by a still incomplete mechanistic disease concept and, therefore, a rudimentary understanding of targetable vulnerabilities in T-PLL. The most potent single substance, the CD52-antibody alemtuzumab, leads to complete remissions in more than 80% of patients, however, nearly all eventually relapse (at median already within 12 months) [3,4,7].

Due to the limited number of primary patient samples available, genomic analyses studying the underlying disease mechanisms have been performed in rather small cohorts of T-PLL patients. In these studies, the most common molecular features revealed were rearrangements that involve chromosome 14 or X, resulting in juxtaposition of the *T-cell leukemia/lymphoma 1A (TCL1A)* or *Mature T-cell leukemia 1 (MTCP1)* proto-oncogenes to *TCRAD* gene enhancer elements [8]. The second most common lesions are genomic alterations of the tumor suppressor *ataxia telangiectasia mutated (ATM)*, found in >85% of cases [9]. In addition to these lesions, activating mutations targeting the Janus kinase (JAK)/signal transducer and activator of transcription (STAT) pathway components were identified in these series of genomic analyses [9,10,11,12,13,14,15,16,17,18].

The JAK/STAT pathway is a ubiquitous cytokine-mediated signaling cascade that regulates cell proliferation, differentiation, migration, and apoptosis [19]. After recruitment of JAKs (JAK1, JAK2, JAK3, TYK2) to cytokine receptors, they phosphorylate STAT proteins (STAT1, STAT2, STAT3, STAT4, STAT5A, STAT5B, STAT6), which then bind DNA and regulate target gene transcription [20]. Dysregulation of the JAK/STAT axis has been described as a key event in the pathogenesis of various hematologic malignancies [21]. Constitutively active JAK/STAT signaling induces T-cell tumors in mice [22,23]. Using different sequencing approaches, activating mutations of *JAK1, JAK3,* and *STAT5B* were identified as the most recurrent genomic aberrations affecting *JAK/STAT* genes in T-PLL [9,10,11,12,13,14,15,16,17,18]. However, prevalence of gene mutations, information on their allele frequencies, assessment of negative regulators of JAK/STAT signaling, and the phosphorylation status of the most recurrently affected JAK/STAT proteins vary considerably or are not reported in these studies [9,10,11,12,13,14,15,16,17,18].

Therapeutic approaches blocking JAK/STAT signaling have so far improved patient outcomes predominantly in autoimmune conditions and in graft-versus-host disease [24,25]. JAK inhibitors are currently tested for a number of new indications [26]. T-PLL cells have shown a notable in vitro sensitivity towards JAK inhibition, which was not directly linked to the *JAK/STAT* mutation status [15,16]. First reports present individual clinical activity of tofacitinib (pan JAK inhibitor) and ruxolitinib (JAK 1/2 inhibitor) in relapsed T-PLL [27,28].

Although many studies identified *JAK* and *STAT* genes to be commonly mutated in T-PLL, these analyses have been performed in rather small cohorts not providing a sufficient dataset to determine reliable mutation and variant allele frequencies (VAFs). In addition, the publication overlap of these studies was unresolved and a systematic assessment for other potential genomic causes (e.g., copy number alterations (CNAs)) has not been performed.

Here, we conducted a meta-analysis that was supplemented by new primary data, hence providing the largest cohort to date that evaluated the genomic aberrations affecting *JAK/STAT* signaling in T-PLL. In addition to summarizing information on the functional impact of the most recurrent lesions, we propose a model of potential mechanisms leading to constitutive JAK/STAT signaling in T-PLL cells.

## 2. Results

### 2.1. Characteristics and Overlaps of Included Studies

**Figure 1:**
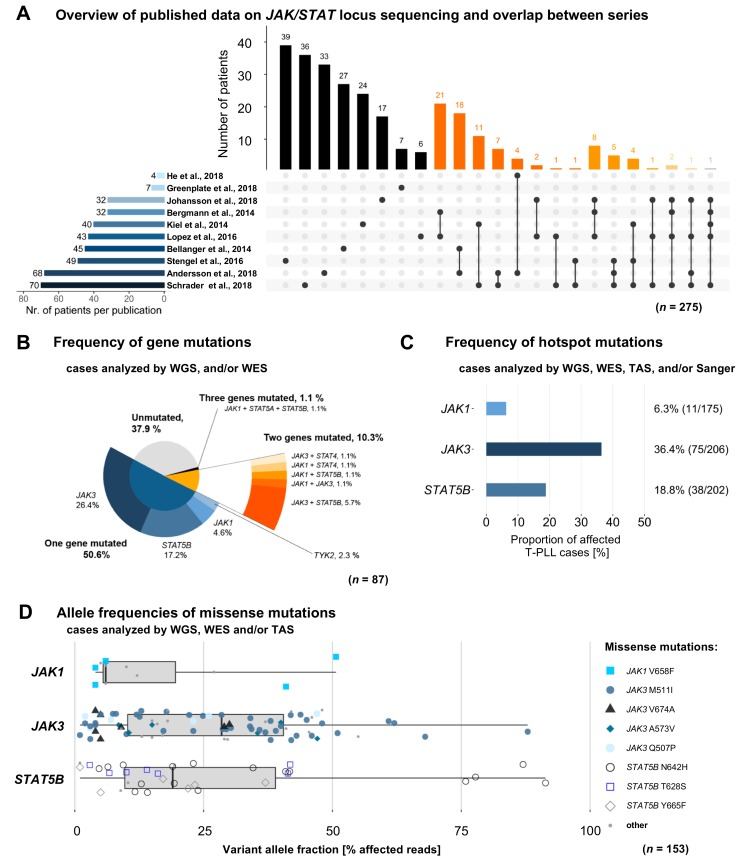
Meta-analyses of genomic profiling series in T-PLL underscore the high prevalence of mutations affecting *JAK* and *STAT* genes. (**A**) T-PLL patients (*n* = 275) sequenced for any *JAK* or *STAT* locus. Horizontal bar chart displays the total number of patients sequenced in each publication. Vertical bar chart indicates the size of intersections between sets of patients analyzed in one or more publications. Color-code of vertical bars indicates the number of studies reporting results of the same individual case (black: 1; dark-orange: 2; medium-orange: 3; light-orange: 4; light grey: 5). Dot connecting lines show the overlapping publications of each intersection. (**B**) Distribution of *JAK/STAT* mutations (*n* = 87 T-PLL analyzed by whole genome sequencing (WGS)/whole exome sequencing (WES)): in 62.1% of these cases (*n* = 54) at least one mutation in a gene of the *JAK/STAT* family was found to be mutated. (**C**) Relative frequencies of hotspot mutations of *JAK1*, *JAK3*, and *STAT5B* (as defined by the genomic region containing more than 90% of the respective mutations; *n* = 275 cases analyzed by WGS/WES/targeted amplicon sequencing (TAS)/Sanger sequencing). Every T-PLL case that was sequenced for the respective hotspot region of *JAK1* (Ex. 14–20), *JAK3* (Ex. 11–19), and/or *STAT5B* (Ex. 15–17), was included. (**D**) Box-whisker charted allele frequencies of missense mutations of *JAK1*, *JAK3*, and *STAT5B* (*n* = 153 T-PLL analyzed by WGS/WES and/or TAS) illustrate a high heterogeneity in variant allele fractions, with most lesions detected at lower frequencies (<50%).

The meta-analysis considered all available publications that have analyzed variants of any *JAK* or *STAT* gene in cases of T-PLL, regardless of the sequencing approach used (Table S1). Redundantly sequenced cases were identified to eliminate overlaps between these 10 studies (Figure 1A). The most common sequencing approach was Sanger sequencing (Sanger seq., 7 studies), followed by targeted amplicon sequencing (TAS, 5 studies), whole exome sequencing (WES, 4 studies), and whole genome sequencing (WGS, 2 studies). *JAK3* (*n* = 272 T-PLL patients), *JAK1* (*n* = 246), and *STAT5B* (*n* = 209) were predominantly sequenced due to the bias by the targeted approaches. Germline controls were sequenced in 53 cases (19.3%). The number of analyzed patients varied from 3 to 71 patients across the 10 studies [9,10,11,12,13,14,15,16,17,18]. After subtracting all cases reported in more than one study, we identified 275 unique T-PLL cases as the core cohort.

Notably, data that were acquired by WGS, WES, and SNP array analysis were re-analyzed as primary data in cases, in which raw data were available (WES: 62.2%, *n* = 46/74 T-PLL patients; WGS: 50.0%, *n* = 4/8; SNP arrays: 100.0%, *n* = 71/71), while data gained through Sanger seq. or TAS were not re-evaluated. By re-analyzing the WES and SNP array data through a uniform pipeline, homogeneity of the resulting dataset was obtained. *JAK1*, *JAK2*, *JAK3*, *TYK2*, *STAT1*, *STAT2*, *STAT3*, *STAT4*, *STAT5A*, *STAT5B*, and *STAT6* are further referred to as ‘any *JAK* or *STAT* gene’.

### 2.2. Mutations in JAK and STAT Genes are Predominantly Found at Subclonal Levels

We included T-PLL cases with available data from WGS or WES in order to calculate the frequency of genomic variants in *JAK* and *STAT* genes. We identified a cumulative rate of 62.1% (*n* = 54/87, Figure 1B) cases with variants in any *JAK* or *STAT* gene. The vast majority of them were somatic single-nucleotide variants (SNVs) of missense type (96.0%, *n* = 72/75 of variants detected by WGS/WES). The remaining three lesions (4.0%, *n* = 3/75) were small in-frame deletions in *JAK1* or *JAK3*. In the following these SNVs and small in-frame deletions are referred to as ‘mutations’. Somatic variants were called and known single nucleotide polymorphisms were filtered out in those tumor samples for which a matched germline sample was available. For T-PLL patients with a missing germline control (*n* = 38), we included only those variants which were detected in the WES with matched germline samples. *JAK3* (34.5%, *n* = 30/87), *STAT5B* (25.3%, *n* = 22/87), and *JAK1* (9.2%; *n* = 8/87) were the most recurrently mutated genes, while *TYK2* (2.3%, *n* = 2/87), *STAT4* (2.3%, *n* = 2/87), and *STAT5A* (1.1%, *n* = 1/87) were mutated at lower frequencies. Nine of 87 cases (10.3%) presented with mutations affecting two genes that encode for JAK or STAT proteins. Co-occuring (‘double’) *JAK1* and *JAK3* mutations were found in 1.1% of cases (*n* = 1/87), *JAK3* and *STAT4* in 1.1% (*n* = 1/87), and *JAK1* and *STAT4* mutations in 1.1% (*n* = 1/87); *JAK1* and *STAT5B* in 1.1% (*n* = 1/87), and *JAK3* and *STAT5B* in 5.8% (*n* = 5/87). Co-occuring *JAK1*, *STAT5A*, and *STAT5B* mutations (‘triple’) were detected in one case (1.1%).

Next, we calculated frequencies of missense mutations considering the hotspots of *JAK1* (Ex. 14–20), *JAK3* (Ex. 11–19), and *STAT5B* (Ex. 15–17). Hotspot regions were defined as genomic regions containing >90% of the respective mutations. By also including cases analyzed by TAS or Sanger seq. for the respective hotspot region, we arrived at a larger cohort for these analyses. Based on this, the hotspot of *JAK1* showed a relative mutation frequency of 6.3% (*n* = 11/175). For the hotspot of *JAK3* this was 36.4% (*n* = 75/206) and for the hotspot of *STAT5B* it was 18.8% (*n* = 38/202, Figure 1C).

As already described in smaller cohorts [9,16,18], most of these lesions presented as subclonal events with low VAFs (medians: *JAK1* 6.0%, *JAK3* 28.5%, *STAT5B* 19.0%, Figure 1D); however, we observed a high heterogeneity in VAFs. Notably, the N642H mutation of *STAT5B* was observed at a VAF of >50% in four cases and the M511I mutation of *JAK3* at VAFs >50% in five cases. Note that given the data provided in the reports, the percentage of treatment-naïve samples was 72.7%; sequential samples of individual T-PLL patients were not analyzed here.

### 2.3. Missense Mutations in JAK1, JAK3, and STAT5B Cluster Within the Conserved Pseudokinase and SH2 Domains

**Figure 2:**
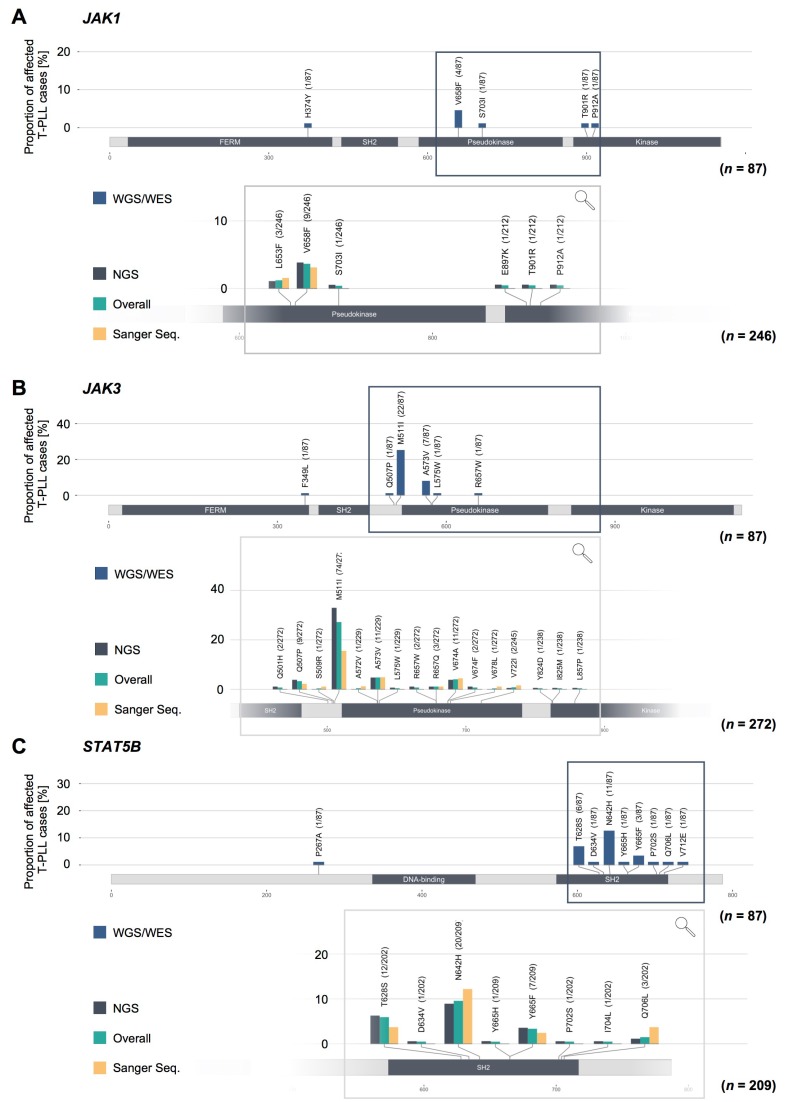
Missense mutations in *JAK1*/*JAK3*/*STAT5B* genes cluster within the conserved JH2 (pseudokinase) and SH2 domains. Upper protein scheme: gene-wide distribution of missense mutations of *JAK1* (**A**), *JAK3* (**B**), and *STAT5B* (**C**); *n* = 87 T-PLL analyzed by WES/WGS, representing the same cases as in Figure 1B. Lower inset for each sub-panel A–C: frequencies of hotspot mutations, stratified for applied sequencing methods (*n* = 275 cases analyzed with WES/WGS/TAS/Sanger seq). While *JAK1* and *JAK3* were predominately affected by lesions within the pseudokinase domains (JH2) and the SH2-JH2 linker regions, *STAT5B* missense mutations were presented mostly in the SH2 domain.

We further assessed the localization of the missense mutations of *JAK1*, *JAK3*, and *STAT5B* (Figure 2, upper protein schemes) as well as the frequencies of hotspot SNVs in these genes (lower inset). To assess the localization of the somatic SNVs of *JAK1*, *JAK3*, and *STAT5B*, we included all T-PLL cases which have been sequenced for the whole coding genome by WES or WGS. All T-PLL cases which have been sequenced for the respective lesion regardless of the sequencing approach were considered to determine the frequencies of hotspot mutations. The pseudokinase domain (JH2 domain) of *JAK1* was predominantly affected (62.5% of all *JAK1* mutations, *n* = 5/8) with V658F (3.7% of all analyzed cases, *n* = 9/246, regardless of the sequencing approach) as the most prominent lesion (Figure 2A). In *JAK3*, most somatic SNVs were found in the JH2-SH2-linker (69.7% of all *JAK3* missense mutations, *n* = 23/33 detected missense mutations), followed by the JH2 domain (27.3%, *n* = 9/33, Figure 2B). The M511I lesion was the most prevalent lesion in *JAK3* (27.2% of all analyzed cases, *n* = 74/272, regardless of the sequencing approach). Almost all missense mutations of *STAT5B* were found in the SH2 domain (96.2% of all *STAT5B* missense mutations, *n* = 25/26 detected missense mutations; Figure 2C). N642H (9.6% of all analyzed cases, *n* = 20/209, regardless of the sequencing approach), T628S (5.9%, *n* = 12/202), and Y665F (3.3%, *n* = 7/209) were the most recurrent mutations affecting *STAT5B*. Remarkably, we did not detect significant differences in the mutation frequencies with regard to the sequencing approach (see bars of the lower inset of Figure 2).

### 2.4. T-PLL Harboring any JAK or STAT Mutation Show Elevated TCL1A mRNA Level, but Present Comparable Clinical Outcomes

**Figure 3:**
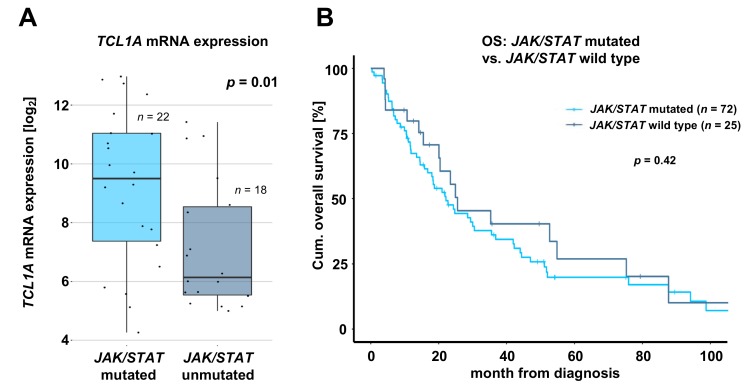
*JAK*/*STAT* mutation status shows association with elevated TCL1A mRNA expression, but not with patient outcomes. (**A**) TCL1A mRNA expression measured by array-based gene expression profiling (GEP) of *JAK*/*STAT* mutated cases compared to cases without any *JAK* or *STAT* mutation (fold change: 4.1; p = 0.01, Student’s *t*-test). (**B**) Overall survival (OS) of *JAK*/*STAT* mutated cases (median OS: 21.0 months) compared to cases without any *JAK* or *STAT* mutation (median OS: 25.5 months, p = 0.42, log rank test). Significant associations between mutations affecting *JAK3* or *STAT5B* with immunophenotypic, mutational, and expression data are displayed in Figure S1. Associations of mutations affecting *JAK1*, *JAK3*, or *STAT5B* with OS are presented in Figure S2.

When analyzing associations of *JAK* and *STAT* mutations with immunophenotypic and cytogenetic data as well with the 18 most prevalent mutations found in T-PLL (as described by Schrader et al. [9]), we detected a decreased proportion of T-PLL cells with CD40L expression (p = 0.05, Fisher’s exact test, Figure S1A) and an increased proportion of T-PLL cells harboring *NOTCH2* mutations (p = 0.03, Fisher’s exact test, Figure S1B) in the *JAK3* mutated cohort (overview of tested associations is provided in Table S2). Notably, T-PLL with any *JAK* or *STAT* mutation showed elevated TCL1A mRNA levels as compared to patients without any *JAK* or *STAT* mutation (fold change (Fc) = 4.1, p = 0.01, Student’s *t*-test, Figure 3A), further underlined by an increased proportion of cases harboring an inversion 14 (q11q32) in the *STAT5B* mutated cohort (p = 0.03, Fisher’s exact test, Figure S1C). TCL1A mRNA levels were also elevated by just considering *JAK3* mutated cases (Fc = 6.5, p = 0.002, Student’s *t*-test, Figure S1D) or *STAT5B* mutated cases (Fc = 3.6, p = 0.02, Student’s *t*-test, Figure S1E) compared to T-PLL without either mutation, respectively. To assess the functional role of variants in *JAK* or *STAT* genes in T-PLL patients, we screened for significant associations between somatic *JAK*/*STAT* SNVs and clinical data, including outcome. For these analyses, we merged clinical information from nine studies [9,10,11,12,13,14,15,16,18]. As not all considered publications reported the same set of parameters, cohort sizes varied between the different analyses. When investigating an association of the *JAK*/*STAT* mutation status with overall survival, we did not observe differences in OS of T-PLL patients harboring any *JAK* or *STAT* mutation compared to patients without *JAK* or *STAT* mutation (p = 0.42, log rank test, Figure 3B). In contrast to studies of smaller cohorts [12,16], we could not observe a significantly shorter OS of *JAK3* mutated T-PLL patients (p = 0.26, log rank test, Figure S2A). Similarly, the *JAK3* M511 mutation was not associated with a shorter OS (p = 0.39, log rank test, Figure S2B). The OS of patients without any *JAK* or *STAT* mutations was comparable to those harboring a *STAT5B* (p = 0.55, log rank test, Figure S2C), *STAT5B* N642H (p = 0.60, log rank test, Figure S2D), or *JAK1* (p = 0.54, log rank test, Figure S2E) mutation. Interestingly, the *JAK1* L653F mutation status was associated with a significantly shorter OS (p = 0.003, log rank test, Figure S2F). In addition, cases with a *STAT5B* Y665F mutation also showed a shorter OS (p = 0.06, log rank test, Figure S2G). However, only two patients with a *JAK1* L653F mutation or five patients with a *STAT5B* Y665F mutation were included in the ‘mutated’ cohorts, so these results should be interpreted with caution. We did not detect any other association of clinical characteristics of T-PLL patients with the presence of *JAK* or *STAT* mutations (Table S3).

### 2.5. T-PLL Cells Show Basal STAT5B Phosphorylation, Regardless of Their JAK/STAT Mutation Status

**Figure 4:**
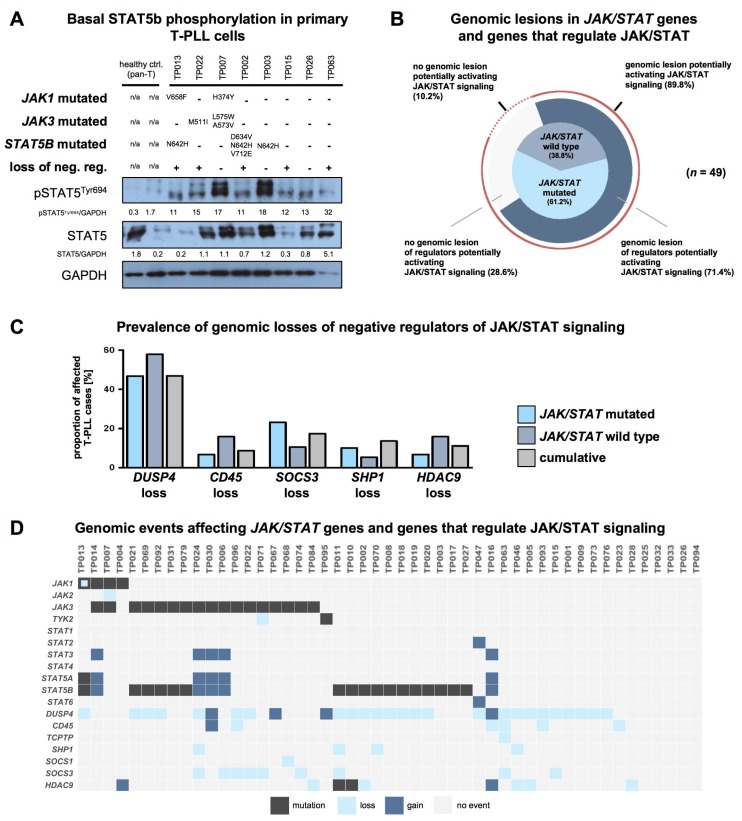
T-PLL cells show basal STAT5B phosphorylation, regardless of their *JAK/STAT* mutation status. (**A**) Elevated basal STAT5B phosphorylation levels of T-PLL cells. Controls: CD3^+^ pan T-cells isolated from healthy individuals. ‘Loss of neg. reg.’: key regulators which negatively affect STAT5B activation (namely *DUSP4, CD45, TCPTP, SHP1, SOCS1*, *SOCS3,* and *HDAC*; see (D) for case-wise depiction) were considered based on available literature and based on data of their copy number alterations (CNA) in T-PLL. (**B**) Distribution of genomic lesions affecting any *JAK* or *STAT* gene and their regulators (*n* = 49 cases analyzed with WES/WGS and with SNP arrays). Inner pie chart: distribution of *JAK/STAT* mutations. Outer pie chart: Prevalence of genomic lesions of regulators activating JAK/STAT (either genomic losses of negative regulators or genomic gains of positive regulators). An overall proportion of 89.8% of T-PLL cases carried a genomic lesion potentially explaining constitutive STAT5B activation (mutation or CNA of JAK/STAT regulator). (**C**) Prevalence of the five most common genomic lesions affecting negative regulators of the JAK/STAT pathway (*n* = 49 cases analyzed with WES/WGS and SNP array): T-PLL cases without any mutation in a *JAK* or *STAT* gene showed a higher prevalence of genomic losses of *DUSP4, CD45,* and *HDAC9* as compared to *JAK/STAT* mutated cases. (**D**) Mapped genomic events involving *JAK* and *STAT* genes and their regulators (*n* = 49 cases analyzed with WES/WGS and SNP array). An overview of genomic lesions resulting in a suggested activation of the JAK/STAT pathway across all considered T-PLL cases is given in Figure S3.

Even though STAT5B was shown to be predominantly affected in T-PLL in previous series, its phosphorylation, and therefore activation state, was often not aligned with the genomic profiling data [9,10,11]. By analyzing primary samples of eight T-PLL cases, we observed noticeable basal STAT5B phosphorylation in every case (Figure 4A, no exposure to any culturing). We discovered no obvious association between STAT5B phosphorylation and the presence of any *JAK* or *STAT* mutation.

Samples without *JAK* or *STAT* mutation showed similar levels of high basal phospho-STAT5B to those that harbored such lesions, as already previously shown in a series of 6 T-PLL cases [9].

We further correlated the immunoblot data with alterations involving key regulators of STAT5B activity (either mutations or CNAs). For this, we pre-selected a set of 105 genes encoding for proteins known to regulate JAK/STAT signaling based on available literature (Table S4). We next identified of these 105 genes 7 which showed recurrent genomic lesions in the overall T-PLL cohort and which were reported to have a regulatory effect on STAT5B signaling in vitro or in vivo (Table S5). Overall, we identified recurrent mutations or genomic losses of seven negative regulators potentially leading to constitutive STAT5B activity (*DUSP4, CD45, TCPTP, SHP1, SOCS1, SOCS3*, and *HDAC9*) in 71.4% of analyzed T-PLL cases (*n* = 35/49 cases analyzed with WES/WGS and SNP array, Figure 4B). About 68.4% of T-PLL cases that did not reveal a mutation in any *JAK* or *STAT* gene showed such a CNA of a STAT5B signaling regulator. This resulted in a total of 89.8% of T-PLL cases that harbor a genomic lesion potentially explaining constitutive STAT5B signaling (either genomic losses of negative regulators, and/or mutations of *JAK* or *STAT* genes). Gene expression signatures (array-based) of cases that harbor a genomic loss or a mutation of a negative regulator of JAK/STAT signaling (and that lack a *JAK* or *STAT* mutation) were comparable to those of cases with a mutation in any *JAK* or *STAT* gene (principal component analysis in Figure S4A). Both cohorts showed similar activation (as per upregulated gene expression) of STAT5B target genes. All T-PLL with a mutation in a *JAK* or *STAT* gene and 90% of patients with a mutation or loss in a negative JAK/STAT regulator (and without any mutation in a *JAK* or *STAT* gene) showed overexpression of at least one STAT5 target gene. In contrast, the frequency of overexpressed STAT5 target genes was significantly lower in T-PLL without a genomic lesion potentially activating JAK/STAT signaling (60%, Figure S4B). In detail, *DUSP4*, *CD45*, *SOCS3*, *SHP1*, and *HDAC9* showed the highest prevalences of genomic losses with a higher frequency in T-PLL cases without any *JAK* or *STAT* mutation (Figure 4C). In addition, we identified genomic gains of *STAT2* (*n* = 1/49 T-PLL), *STAT3*, (*n* = 5/49 T-PLL), *STAT5A* (*n* = 5/49 T-PLL), and *STAT5B* (*n* = 5/49 T-PLL). These lesions were detected only in combination with mutations of a *JAK* or *STAT* gene or with genomic losses of a negative regulator of STAT5B signaling (Figure 4D).

Overall, we propose a model of constitutive JAK/STAT signaling induced by mutations of a predicted gain of function (GOF) in *JAK1*, *JAK3*, and *STAT5B* genes and by copy-number losses of negative regulators of STAT5B activity.

## 3. Discussion

Here, we present a comprehensive meta-analysis of primary data from 275 T-PLL cases from 10 published studies that evaluated genomic lesions involving elements of JAK/STAT signaling. In this large cohort, we identified genomic aberrations potentially leading to constitutive JAK/STAT signaling in approximately 90% of cases, thus establishing this as a molecular hallmark of T-PLL. Our analysis provides an overview on the genomic causes of JAK/STAT signaling activation including *JAK/STAT* GOF mutations as well as CNAs of *JAK/STAT* genes or negative regulators of JAK/STAT signaling.

About 62% of T-PLL cases showed GOF mutations affecting any *JAK* or *STAT* gene, while previous publications reported a mutation frequency varying between 36% and 76% [10,18]. In accordance with these studies, we predominantly detected missense mutations in the SH2 or pseudokinase domains of *JAK1*, *JAK3,* and *STAT5B*, mostly occurring at a subclonal level. Mutations of genes encoding for components of the JAK/STAT pathway are frequently found in T-cell neoplasms (predominantly mutations of *JAK3*, *STAT3,* and *STAT5B*). Their reported incidences range from 10% (e.g., for Sézary syndrome [29]) to 70% (e.g., for intestinal T-cell lymphoma [30]). Compared to those, the cumulative mutation frequency of *JAK* and *STAT* genes in T-PLL documented in this meta-analysis is high [31].

Another important finding was the high basal phosphorylation of STAT5B in all analyzed T-PLL samples. In addition to a high proportion of T-PLL cases mutated in any *JAK* or *STAT* gene, we identified seven negative regulators to be commonly lost in T-PLL, potentially explaining cytokine-independent STAT5B activation. This affected 71.4% of all cases and 68.4% of those cases without a detectable *JAK/STAT* SNV. Of these negative regulators, *DUSP4*, *SOCS3*, and *CD45* were most frequently affected. While genomic losses of *DUSP4* and *SOCS3* were already reported in primary T-PLL [9,17,18], losses of *CD45* have not been described previously. Compared to CD4^+^ T-cells isolated from healthy individuals, DUSP4 and CD45 mRNA was observed to be strongly downregulated in T-PLL cells [16]. Furthermore, the downregulation of CD45 transcripts was associated with an increased phosphorylation of STAT5, implicating a functional relevance of this negative regulator [17].

T-PLL cells usually show a complex karyotype and a high frequency of somatic CNAs [9,32]. In detail, the loci of the negative regulators *DUSP4* (chr.8p12) and of *SOCS3* (chr.17q25.3) are known to be highly affected by structural aberrations in T-PLL. Therefore, a secondary origin of the detected CNAs due to background imbalances has to be considered. T-PLL cases already harboring an activating mutation of a *JAK* or *STAT* gene or a genomic loss of a negative regulator showed in few cases (12.2%) also genomic gains of *JAK* or *STAT* genes, however, the functional impact of these genomic gains remains unclear.

Certainly, there is the possibility of other, yet-unknown regulators, which we did not include in our analysis. Furthermore, our analysis did not assess for epigenetic regulations or post-translational modifications of *JAK* and *STAT* genes or their regulators, as further causes of active JAK/STAT signaling. Moreover, constitutive T-cell-receptor or cytokine input of the malignant T-cell may represent other modes of (milieu-derived) JAK/STAT activation in T-PLL.

Another important result of the provided meta-analyses is the first description of elevated TCL1A in *JAK* and *STAT* mutated T-PLL. TCL1A overexpression is known to be associated with elevated levels of reactive oxygen species (ROS) [33]. In addition, an 8-oxoguanine DNA damage signature is characteristic for T-PLL, likely resulting from ROS-mediated DNA insults [9]. In this context, the initiating oncogene TCL1A is likely to contribute to the occurrence of new mutations, especially when the guardian of genome-integrity *ATM* is deleted or mutated, potentially explaining a higher prevalence of arising subclonal *JAK* and *STAT* mutations in T-PLL. Furthermore, a possible explanation for the association of reduced CD40L expression with the presence of *JAK3* mutations is a state of less T-cell receptor and cytokine dependent activation of T-PLL cells in the context of an acquired hyperactivating mutation of a signaling intermediate such as *JAK3*.

In line with our finding, that nearly every T-PLL case presents with a genomic aberration that potentially causes activated JAK/STAT signaling, we could not observe any differential association of the *JAK/STAT* mutation status with clinical characteristics, i.e., those indicating a more aggressive phenotype of T-PLL cells. Previous studies on small cohorts have shown a negative influence of the presence of *JAK3* mutations on OS [12,16]. However, as these studies used *JAK3* wild type T-PLL as a control group, they potentially included patients with mutations of other *JAK* or *STAT* genes, therefore not representing a proper control.

Although functional validations of the herein described genomic aberrations is outside the scope of this work, mechanistic analyses of many of the detected genomic aberrations and of their impact on tumorigenesis have been performed in various other entities. Such functional findings of mutations of *JAK1*, *JAK3,* and *STAT5B* are summarized in the following (an overview of literature is provided in Table S5).

*JAK1*, the third most frequently mutated *JAK* or *STAT* gene in this meta-analysis, showed a cumulative hotspot mutation rate of 6.3% in our analysis, in line with the mutation rates calculated in previous studies. V658F, the most prevalent lesion affecting *JAK1* in T-PLL, was initially described in the development of T-ALL [34]. The V658F lesion is homologous to the *JAK2* V617F mutation and was shown to lead to constitutive active JAK1 signaling in a cell line model [35]. Cells harboring the respective lesion presented enhanced basal JAK1, STAT5, and ERK phosphorylation and showed cytokine-independent growth [36]. S7031 and L653F, less frequently detected *JAK1* mutations, were also proven to act as GOF mutations [37,38]. While the S703I mutation was shown to mediate proliferative effects in vitro and in vivo, the L653F mutation is predicted to soften the negative regulation of JAK1 in a biophysical model. The *JAK1* V617F, S703I, and L653F mutations potentially mediate cytokine-independent phosphorylation of STAT5B in T-PLL.

*JAK3* mutations were the most recurrent genomic aberration affecting *JAK/STAT* genes in this meta-analysis with a hotspot mutation rate of 36.4% (literature: 21% [12]–71% [17]). The oncogenic potential of the most frequent *JAK3* mutation M511I as well as the less frequently occurring mutations A573V and V674A was shown in various systems: M511I, A573V, as well as V674A mutant cell lines demonstrated high phosphorylation of STAT5 and ERK, leading to cytokine independent growth [39,40]. When transplanting mice with bone marrow progenitor cells harboring the M511I or A573V mutation in *JAK3*, they develop a T-ALL-like disease, characterized by an expansion of immature CD8^+^ T-cells. In contrast, mice transplanted with V674A mutant bone marrow progenitor cells presented severe lymphadenopathy and splenomegaly without a significant increase of the WBC count [41]. The mentioned *JAK3* mutations were also described in T-ALL [42]. The Q507P mutation, the third most frequent genomic aberration affecting *JAK3* in T-PLL, was not discovered in other entities and has so far not been investigated for its functional relevance.

In this analysis, 18.8% of T-PLL cases were mutated within the hotspot region of *STAT5B*, while the mutation rate in the literature varied from 7% [16] to 36% [10]. The most frequently found N642H mutation is a well described GOF mutation: *STAT5B* N642H transduced cell lines showed prolonged phosphorylation and dimerization of STAT5B, resulting in an enhanced transcriptional activity [43,44]. The N642H mutation leads to a sustained stability to the anti-parallel dimer as shown by crystallization [45]. Transgenic mice harboring the *STAT5B* N642H lesion rapidly developed aggressive CD8^+^ T-cell neoplasms [46]. The *STAT5B* Y665F mutation, which had a notable influence on OS of T-PLL patients in our analysis, revealed highly phosphorylated STAT5B, an enhanced transcriptional activity and increased proliferation in a cell culture model, comparable to the *STAT5B* N642H mutation. The above-mentioned genomic aberrations, which have also been described in the mutational landscape of T-LGL [47], may play an important role in the activation of JAK/STAT signaling in T-PLL. The functional role of the second most detected *STAT5B* aberration, the T628S mutation, is much less established. This genetic aberration is previously described in hepatosplenic T-cell lymphoma and associated with an increased phosphorylation of STAT5 in a cell line model [48]. Notably, the three commonly affected *JAK/STAT* genes (*JAK1, JAK3, STAT5B*) are crucial for IL-2 signaling, the cytokine secreted at highest levels in T-PLL [49]. Targeted approaches in appropriate models have to address the chronological role of such lesions as most of them were detected at subclonal levels.

Given that nearly every T-PLL case harbors a genomic aberration potentially activating JAK/STAT signaling, JAK and STAT proteins are clinically relevant targets for T-PLL therapy [50]. Interestingly, in a large panel of blood cancers, primary T-PLL patient cells showed high sensitivity towards JAK-targeting compounds. Moreover, JAK inhibitors were among the top 25 most effective drugs in an *ex vivo* drug screen of 39 T-PLL patient samples exposed to 301 different substances [15,16]. Previous case reports also showed the initial promising results of a combination therapy of the JAK inhibitors ruxolitinib (JAK 1/2 inhibitor) and tofacitinib (pan JAK inhibitor) [27,28]. However, previous studies have also argued that the *JAK/STAT* mutation status alone does not predict ex-vivo sensitivity towards JAK inhibitors [16]. This is in line with the observation of multiple, potentially distinct mechanisms, apart from missense mutations of *JAK/STAT* genes, that are required for activating this pathway. In general, predicting sensitivity of kinase inhibitors often requires larger panels of biomarkers, some of which may be outside of the target pathways or direct drivers of the disease. Therefore, therapeutic targeting of JAK/STAT signaling has to be expanded on various levels, including the development of novel STAT3/5 inhibitors and the extension of pre-clinical and clinical studies to large cohorts of T-PLL and other T-cell lymphomas, all with well-characterized tumor material (e.g., JAK/STAT activation state). Selective STAT5 inhibitors which bind to the SH2 domain of *STAT5* are currently under development [51].

## 4. Materials and Methods

### 4.1. Data Acquisition

Available literature was systematically screened for all genomic profiling studies that have information on the mutational status of any *JAK* or *STAT* gene in T-PLL, regardless of the sequencing technique. We identified a total of 10 publications meeting these criteria (Table S6). Raw data of these publications was obtained from different sources, either publicly available or kindly provided by the corresponding authors. Full descriptions of the composed data set and detailed information on the sources of data are provided in Table S6. Considered T-PLL patients had provided written informed consent and all the studies were originally approved by their institutional review boards.

### 4.2. Data Merging

Considering the rarity of T-PLL and the strong collaboration network in T-PLL research, we assumed a pronounced exchange of patient samples between different centers and studies. To determine all redundantly analyzed patients in our cohort, we requested basic patient information to allow us to precisely identify potential overlaps while still ensuring anonymity of all the patients (Figure 1A). In cases where genomic regions were sequenced by different methods, we included the information provided by the widest type of analysis, ranking WGS > WES > TAS > Sanger sequencing. In total, 275 distinct T-PLL patients were included in our analyses.

### 4.3. Raw Data Analysis

Data acquired through whole-exome sequencing and SNP-array analysis was re-analyzed for those cases in which raw data was available, while data obtained through Sanger sequencing or targeted amplicon sequencing was not re-analyzed. Studies where raw data was not available were included by applying the already analyzed data. Detailed information on the data sources are listed in Table S6 and on the applied tools with references in Table S7. The below subsections describe the various genomic analyses performed for the meta-analysis.

#### 4.3.1. Whole-Exome Sequencing (WES)

The sequenced raw exome reads were trimmed of B blocks from the end using Trimmomatic (RWTH Aachen, Aachen, Germany). The trimmed data were aligned to the human reference genome (GRCh build 37) using Burrows Wheeler Aligner (bwa-0.7.12, Wellcome Trust Sanger Institute, Cambridge, UK). After alignment the potential PCR duplicates were removed using Picard (Broad Institute of Harvard and MIT, Cambridge, MA, USA), and BAM files were generated, sorted, and indexed using SAMtools (Wellcome Trust Sanger Institute, Cambridge, UK). Exomic regions were re-aligned and the base quality scores were re-calibrated according to the Genome Analysis Toolkit Best Practices recommendations (GATK 4.1.3.0). VarScan2.2.3 (The Genome Institute, St. Louis, MO, USA) was used to call somatic mutations with the following parameters: strand-filter 1, min-coverage-normal 8, min-coverage-tumor 6, somatic-*p*-value 0.01, min-var-freq 0.05. The putative somatic mutations were annotated for functional consequences using SnpEff 4.03 (Institute of Environmental Health Sciences, Detroit, MI, USA). Variants present in 1000 genome or dbSNP 2.0 Build were filtered out from the analysis.

In order to call somatic single-nucleotide variants in 38 patients without matched germline samples, MuTect v2 (Broad Institute of Harvard and MIT) was employed with default parameters. Because we were mostly interested in identifying sequence variants that were likely to contribute to T-PLL pathogenesis, we focused on those variations that were identified in our analysis of 22 patients with matched healthy control samples. Only these filtered mutations have been reported for the patients without matched germline samples.

#### 4.3.2. Somatic Copy-Number Alterations (sCNAs)

Genotyping of Affymetrix GenomeWide SNP6 array (Thermo Fisher Scientific, Waltham, MA, USA) was performed using Genotype Console Software 4 (GTC4, Thermo Fisher Scientific) with the default parameters using Birdseed v2 algorithms (Broad Institute of Harvard and MIT). In order to infer copy number variations in the T-PLL genome, we used the pooled controls as a reference (non-tumor hematopoietic cell DNA as ‘germline’ from T-PLL patients, *n*  =  13) created using the GTC pipeline. The Canary algorithm was used to call CN state using the default settings in GTC. We performed segmentation analysis on the basis of identified SNPs/CNVs using inbuilt algorithm. Copy number values and segmentation were visually assessed in the Integrative Genomics Viewer (Broad Institute, Cambridge, MA, USA). Since the segmentation algorithm only reports significantly altered segments/regions, we mapped identified genomic regions to genes based on version 75 of the Ensembl annotation using BiomaRt (version 2.38 and the GenomicRanges R package, version 1.34.0).

#### 4.3.3. Gene Expression Profiling (GEP)

The processed normalized expression profiles generated with GenomeStudio were downloaded from GEO. Data were merged and adjusted for batch effects using linear regression. A differential expression analysis was performed using Student’s *t*-test on expression values of patients and healthy controls (CD3+ pan T-cells from 10 healthy donors). The differentially expressed probes were mapped to the respective genes by Illumina provided annotation files. Principal component analysis of the whole gene expression set was performed with R.

### 4.4. Clinical Data and Statistical Analyses

All publications were screened for accompanying clinical data and authors were contacted for further information. In total, we obtained and merged clinical data from nine publications [9,10,11,12,13,14,15,16,18]. For patients who were reported in multiple studies, we included the most detailed clinical data set. To assess possible correlations between mutational status and clinical phenotypes, we performed a screening approach testing for a broad range of parameters (Table S3), where the patients were stratified by overall mutational status, mutational status of specific genes, and by specific mutations. As a control, we used clinical data from T-PLL patients, who were unmutated in all *JAK/STAT* genes analyzed with WES/WGS.

All statistical analyses were performed using R software and R packages (Table S7). Analysis of overall survival, as measured from day of diagnosis until day of event or censoring, was performed and graphed with R survival and R survminer packages. Log rank statistics were calculated to test for differences in survival distributions. Continuous clinical parameters were tested for normal distribution with Shapiro–Wilk test and further assessed for their associations to *JAK/STAT* mutational status with either Student’s *t*-test or Wilcoxon rank sum test, depending on the normality test. Fisher’s exact test was used for testing differences in categorical data in patients with *JAK/STAT* mutations compared to unmutated patients. A *p* < 0.05 was considered to be statistically significant.

### 4.5. Immunoblots

We performed Western blots of whole-cell lysates of primary T-PLL cells and CD3+ pan T-cell isolated from age matched healthy controls by magnetic-bead based cell enrichment. Positive selection of CD3+ pan T-cells of healthy controls was performed according to the manufactures instruction (Biolegend). Following antibodies from Cell Signaling Technology were used in a 1:1000 dilution according to the manufacturer’s instruction: phospho-STAT5TYR694 (clone C11C5, catalogue #9359), STAT5 (polyclonal, #9363), and GAPDH (clone 14C10, #2118). As a secondary antibody we made use of HRP-coupled anti-rabbit (Dianova) in a 1:5000 dilution. Immunoblots were done using Western Bright ECL (Advansta, Menlo Park, CA, USA) and luminescence intensities were evaluated by densitometry (ImageJ software, National Institute of Health, Bethesda, MD, USA). Uncropped images of the immunoblots are displayed in Figure S5.

### 4.6. Data Accessibility

All genomic data analyzed in this study was gathered from published series. For detailed sources of raw data and/or information on *JAK/STAT* mutations, see Table S6.

## 5. Conclusions

**Figure 5:**
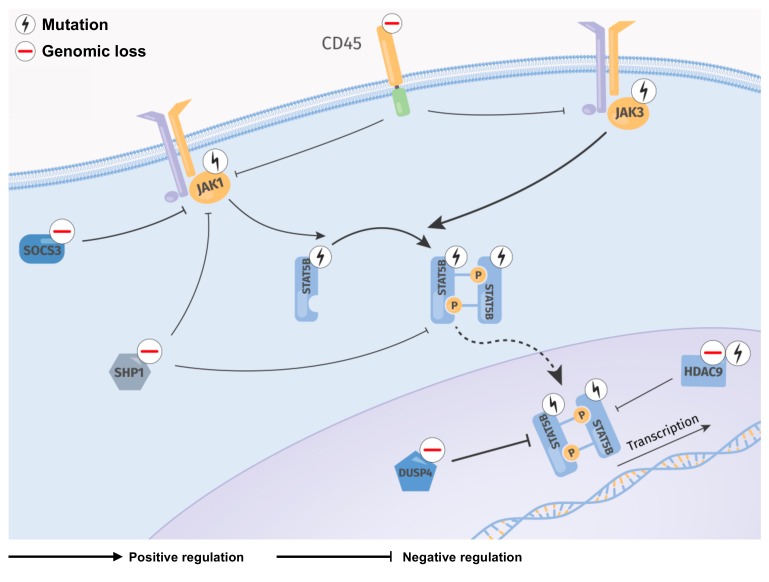
Proposed model of recurrent genomic lesions leading to an enhanced activation of STAT5B in T-PLL. Regulatory network summarizing detected genomic lesions (sCNAs, mutations) in *JAK/STAT* genes and their direct regulators. Mutations of *JAK* and *STAT* genes and genomic losses of negative STAT5B regulators being affected in more than 5% of T-PLL patients were included. Frequent missense mutations occur in the JH2 and SH2-JH2 linker of *JAK1* and *JAK3*, potentially leading to elevated phosphorylation and dimerization of STAT5B. Activation of JAK1 and JAK3 is potentially enhanced through genomic losses (*DUSP4, SOCS1, SOCS3, CD45*, *SHP1, HDAC9*, and *TCPTP*) and mutations (*HDAC9*) of negative regulators. STAT5B activation might be further increased through GOF mutations in its SH2 domain. Cytoplasmatic (*SHP1, TCPTP*) as well as nuclear (*DUSP4, HDAC9*) regulators are commonly affected by genomic losses in T-PLL, leading to intensified STAT5B signaling. Constitutive active STAT5B translocates into the nucleus and regulates transcription of many target genes relevant for T-cell development, differentiation, proliferation, migration, and apoptosis [52].

Overall, this meta-analysis provides an overview of the lesional landscape of *JAK/STAT* associated genes in the largest cohort of T-PLL cases to date and summarizes the potential functional impact of the most common genomic aberrations. Nearly every T-PLL case in our cohort had a genetic aberration, which can be intuitively implicated in constitutively active JAK/STAT signaling. Besides GOF missense mutations in *JAK/STAT* genes, genomic losses of negative regulators of the JAK-STAT signaling axis are frequently found in T-PLL, linking the high basal phosphorylation of STAT5B to genomic causes. We propose a model for these distinct mechanisms implicated in constitutive STAT5B signaling in T-PLL (Figure 5). Our data provide a rational framework for strategies to inhibit JAK/STAT signaling in T-PLL, even in patients whose leukemia does not carry mutations in a *JAK* or *STAT* gene. Development of novel STAT inhibitors and their application, including with synergistic partners, in model systems and in T-PLL patients, represent future tasks.

## Supplementary Materials

The following materials are available online at https://www.mdpi.com/2072-6694/11/12/1833/s1, Figure S1: *JAK/STAT* mutation status shows association with the inversion 14 (q11q32) and elevated *TCL1A* mRNA expression; Figure S2: *JAK/STAT* mutation status shows no association with outcome in T-PLL patients; Figure S3: Overview of genomic lesions associated with an activation of the JAK/STAT pathway across all considered T-PLL cases; Figure S4: T-PLL cases harboring any *JAK* or *STAT* mutation show similar gene expression compared to T-PLL cases with a loss or mutation of a negative regulator potentially activating JAK/STAT signaling; Figure S5: Uncropped images of immunoblots; Table S1: Overview of considered publications; Table S2: Associations of *JAK* and *STAT* mutations with immunophenotypic, cytogenetic, and mutational data; Table S3: Screening of clinical parameters; Table S4: List of genes encoding for proteins which potentially regulate JAK/STAT signaling; Table S5: Overview of references; Table S6: Data sources; Table S7: Bioinformatic tools.
