# Supplementary material for: JAK/STAT-Activating Genomic Alterations Are a Hallmark of T-PLL
Source: Cancers (Basel). 2019 Nov 21;11(12):1833. doi: 10.3390/cancers11121833 (PMC6966610; doi:10.3390/cancers11121833)
Supplement: Supplementary file 1 [file cancers-11-01833-s001.zip › supplementary_figures and tables S5 and S7.docx]

**Supplementary Materials: JAK/STAT-Activating Genomic Alterations are a Hallmark of T-PLL**

**Linus Wahnschaffe, Till Braun, Sanna Timonen, Anil K. Giri, Alexandra Schrader, Prerana Wagle, Henrikki Almusa, Patricia Johansson, Dorine Bellanger, Cristina López, Claudia Haferlach, Marc-Henri Stern, Jan Dürig, Reiner Siebert, Satu Mustjoki, Tero Aittokallio and Marco Herling**

**Figure S1.** *JAK/STAT* mutation status shows association with the inversion 14 (q11q32) and elevated TCL1A mRNA expression. (**A***,***B**) Decreased proportion of T-PLL patients with a high CD40L expression (**A**, measured by flow cytometry, *p* = 0.05, Fisher’s exact test) and increased proportion with *Neurogenic Locus Notch Homolog Protein 2* (*NOTCH2*) mutations (**B**, identified by WES/WGS, *p* = 0.03, Fisher’s exact test) in the *JAK3* mutated cohort. **(C)** Increased proportion of T-PLL patients with an inversion 14 (q11q32) in the *STAT5B* mutated cohort (identified by cytogenetics, *p* = 0.03, Fisher’s exact test)*.* (**D**,**E**) TCL1A mRNA expression (measured by gene expression profiling (GEP array) of *JAK3* mutated cases (**D**, fold change: 6.5., *p* = 0.002, Student’s t-test) and of *STAT5B* mutated cases (**E**, fold change: 3.6., *p* = 0.02, Student’s t-test) compared to cases without any *JAK* or *STAT* mutation. The presence of *STAT5B* and *JAK3* mutations is associated with the TCL1A status, while *JAK3* mutations are associated with reduced CD40L expression and a higher proportion of *NOTCH2* mutations.

**Figure S2.** *JAK/STAT* mutation status shows no association with outcome in T-PLL patients. (**A**–**G**) Overall survival (OS) of *JAK3* mutated cases (**A**, median OS: 18.7 months, *p* = 0.26), *JAK3* M511 mutated cases (**B**, median OS: 18.7 months, *p* = 0.29), *STAT5B* mutated cases (**C**, median OS: 21.0 months, *p* = 0.55), *STAT5B* N642H mutated cases (**D**, median OS: 28.5 months, *p* = 0.60), *JAK1* mutated cases (**E,** median OS: 13.3 months, *p* = 0.54), *JAK1* L653F mutated cases (**F**, median OS 7.3 months, *p* = 0.003), and *STAT5B* Y665F mutated cases (**G**, median OS: 7.7 months, *p* = 0.06) compared to cases without any *JAK* or *STAT* mutation (median OS: 25.5 months). The presence of a *STAT5B* Y665F and *JAK1* L653F mutation correlates with a shorter OS.

**Figure S3.** Overview of genomic lesions associated with an activation of the JAK/STAT pathway across all considered T-PLL cases. Heatmap summarizing JAK/STAT activating genomic events across all analyzed patients and methods (*n* = 275 analyzed by WGS/WES/TAS/Sanger seq.). Mutation analysis including patients sequenced for the respective genes hotspot region as previously defined in Figure 1C and Figure 2A–C. Copy number alterations, analyzed by SNP array, were evaluated for their impact on JAK/STAT activation based on available literature. Consequently, mutations and copy number gains in direct members of the *JAK* and *STAT* family members as well as genomic losses of negative regulators were classified as JAK/STAT activating lesions.

**Figure S4.** T-PLL cases harboring any *JAK* or *STAT* mutation show similar gene expression compared to T-PLL cases with a loss or mutation of a negative regulator potentially activating JAK/STAT signaling. (**A**) Array-based gene expression profiling (GEP) on 38 primary T-PLL patients with known mutational and copy number status (analyzed by WGS/WES and SNP array). Unsupervised exploration of gene expression performed by principal component analysis of the whole data sets. T-PLL samples with a mutation or loss in a negative regulator potentially activating JAK/STAT signaling cluster together with T-PLL cases harboring a *JAK* or *STAT* mutation and T-PLL cases without a genomic lesion potentially activating JAK/STAT signaling. (**B**) Array-based gene expression profiling (GEP) on 38 primary T-PLL patients with known mutational and copy number status (analyzed by WGS/WES and SNP array) compared to CD3+ pan T-cells derived from 10 healthy donors. Frequency of overexpression (fold change ≥ 1.5) of STAT5 target genes (based on available literature, namely *BCL2, BCL2L1, CCND1, CDKN1A, CISH, MYC, SOCS1*). All T-PLL patients with a mutation in a *JAK* or *STAT* gene and 90% of patients with a mutation or loss in a negative regulator (and without any mutation in a *JAK* or *STAT* gene) showed overexpression of at least one target gene, while the frequency of overexpressed STAT5 target genes was significantly lower in T-PLL patients without a genomic lesion potentially activating JAK/STAT signaling (60%, *p* = 0.04, Fisher’s exact test). Notably, this analysis has to be interpreted with caution because other regulation mechanisms of the selected genes besides transcriptional activation through STAT5B were described (i.e., genomic gains of chromosome 8q regulating *MYC* expression).

**Figure S5.** Uncropped images of immunoblots.

**Table S5.** Overview of references.

| **Protein/Mutation** | **References** |
| --- | --- |
| Activating missense mutations | |
| *JAK1* L653F | [1] |
| *JAK1* V658F | [2–4] |
| *JAK1* S7031 | [5] |
| *JAK3* M511I | [6–9] |
| *JAK3* A573V | [7,8] |
| *JAK3* V674A | [8] |
| *STAT5B* T628S | [10] |
| *STAT5B* N642H | [11–14] |
| *STAT5B* Y665F | [11,12] |
| Selected negative regulators of JAK/STAT signaling * | |
| DUSP4 | [15,16] |
| CD45 | [17] |
| TCPTP | [18] |
| SHP1 | [19,20] |
| SOCS1 | [21,22] |
| SOCS3 | [23,24] |
| HDAC9 | [25] |

* Selection of 7 genes out of 105 genes encoding for proteins known to regulate JAK/STAT signaling (based on available literature, 105 genes listed in Table 4). Selection criteria: (i) recurrently affected in T-PLL (mutation or copy number alteration) and (ii) reported regulatory effect on STAT5B activity in vitro or in vivo.

**Table S7.** Bioinformatic tools.

| **Name** | **Version** | **1st Author, Year[ref] or Commercial Source** |
| --- | --- | --- |
| **R packages** | | |
| dplyr | 0.8.1 | Wickham, 2019[26] |
| ggplot2 | 3.1.1 | Wickham, 2016[27] |
| R | 3.5.1 | R Core Team, 2018[28] |
| readxl | 1.3.1 | Wickham, 2019[29] |
| survival | 2.44-1.1 | Therneau, 2000[30] |
| survminer | 0.4.6 | Kassambara, 2019[31] |
| svglite | 1.2.2 | Wickham, 2019[32] |
| UpSetR | 1.4.0 | Gehlenborg, 2019[33] |
| xlsx | 0.6.1 | Dragulescu, 2018[34] |
| BiomaRt | 2.38 | Durinck, 2005[35] |
| GenomicRanges | 1.34.0 | Lawrence, 2013[36] |
| **Software** | | |
| Trimmomatic | 0.38 | Bolger, 2014[37] |
| DGV | GRCh build 37 | MacDonald, 2014[38] |
| Burrows Wheeler Aligner | 0.7.12 | Li, 2009[39] |
| Picard |  | DePristo, 2011[40] |
| SAMtools |  | Li, 2009[41] |
| Genome Analysis Toolkit | GATK 4.1.3.0 | McKenna, 2010[42] |
| VarScan2 | 2.2.3 | Koboldt, 2012[43] |
| GenomeStudio Software |  | Illumina© |
| SnpEff | 4.03 | Cingolani, 2012[44] |
| 1000 genome |  | Abecasis, 2012[45] |
| NCBI dbSNP |  | Sherry, 2001[46] |
| MuTect | v2 | Cibulskis, 2013[47] |
| Birdseed v2 |  | Korn, 2008[48] |
| GTC4 Canary algorithm | 4.2 | Thermo Fisher Scientific© Min, 2004[49] |
| Integrative Genomics Viewer |  | Thorvaldsdóttir, 2013[50] |
| Ensembl annotation | Version 75 | Hunt, 2018[51] |

Reference

1. Flex, E.; Petrangeli, V.; Stella, L.; Chiaretti, S.; Hornakova, T.; Knoops, L.; Ariola, C.; Fodale, V.; Clappier, E.; Paoloni, F.; et al. Somatically acquired *JAK1* mutations in adult acute lymphoblastic leukemia. *J. Exp. Med.* **2008**, *205*, 751–8, doi:10.1084/jem.20072182.
2. Jeong, E.G.; Kim, M.S.; Nam, H.K.; Min, C.K.; Lee, S.; Chung, Y.J.; Yoo, N.J.; Lee, S.H. Somatic mutations of *JAK1* and *JAK3* in acute leukemias and solid cancers. *Clin. Cancer Res.* **2008**, *14*, 3716–3721, doi:10.1158/1078-0432.CCR-07-4839.
3. Gordon, G.M.; Lambert, Q.T.; Daniel, K.G.; Reuther, G.W. Transforming JAK1 mutations exhibit differential signalling, FERM domain requirements and growth responses to interferon-γ. *Biochem. J.* **2010**, *432*, 255–265, doi:10.1042/BJ20100774.
4. Hornakova, T.; Staerk, J.; Royer, Y.; Flex, E.; Tartaglia, M.; Constantinescu, S.N.; Knoops, L.; Renauld, J.-C. Acute lymphoblastic leukemia-associated JAK1 mutants activate the Janus kinase/STAT pathway via interleukin-9 receptor alpha homodimers. *J. Biol. Chem.* **2009**, *284*, 6773–6781, doi:10.1074/jbc.M807531200.
5. Yang, S.; Luo, C.; Gu, Q.; Xu, Q.; Wang, G.; Sun, H.; Qian, Z.; Tan, Y.; Qin, Y.; Shen, Y.; et al. Activating JAK1 mutation may predict the sensitivity of JAK-STAT inhibition in hepatocellular carcinoma. *Oncotarget* **2016**, *7*, 5461–5469, doi:10.18632/oncotarget.6684.
6. Degryse, S.; de Bock, C.E.; Demeyer, S.; Govaerts, I.; Bornschein, S.; Verbeke, D.; Jacobs, K.; Binos, S.; Skerrett-Byrne, D.A.; Murray, H.C.; et al. Mutant JAK3 phosphoproteomic profiling predicts synergism between JAK3 inhibitors and MEK/BCL2 inhibitors for the treatment of T-cell acute lymphoblastic leukemia. *Leukemia* **2018**, *32*, 788, doi:10.1038/leu.2017.276.
7. Martinez, G.S.; Ross, J.A.; Kirken, R.A. Transforming mutations of Jak3 (A573V and M511I) show differential sensitivity to selective JAK3 inhibitors. *Clin. Cancer Drugs* **2016**, *3*, 131–137, doi:10.2174/2212697X03666160610085943.
8. Degryse, S.; de Bock, C.E.; Cox, L.; Demeyer, S.; Gielen, O.; Mentens, N.; Jacobs, K.; Geerdens, E.; Gianfelici, V.; Hulselmans, G.; et al. JAK3 mutants transform hematopoietic cells through JAK1 activation, causing T-cell acute lymphoblastic leukemia in a mouse model. *Blood* **2014**, *124*, 3092–3100, doi:10.1182/blood-2014-04-566687.
9. Vicente, C.; Schwab, C.; Broux, M.; Geerdens, E.; Degryse, S.; Demeyer, S.; Lahortiga, I.; Elliott, A.; Chilton, L.; Starza, R. La; et al. Targeted sequencing identifies associations between IL7R-JAK mutations and epigenetic modulators in T-cell acute lymphoblastic leukemia. *Haematologica* **2015**, *100*, 1301–1310, doi:10.3324/HAEMATOL.2015.130179.
10. McKinney, M.; Moffitt, A.B.; Gaulard, P.; Travert, M.; De Leval, L.; Raffeld, A.N.M.; Jaffe, E.S.; Pittaluga, S.; Xi, L.; Heavican, T.; et al. The genetic basis of hepatosplenic T-cell lymphoma. *Cancer Discov.* **2017**, *7*, 369–379, doi:10.1158/2159-8290.CD-16-0330.
11. Rajala, H.L.M.; Eldfors, S.; Kuusanmäki, H.; van Adrichem, A.J.; Olson, T.; Lagström, S.; Andersson, E.I.; Jerez, A.; Clemente, M.J.; Yan, Y.; et al. Discovery of somatic STAT5b mutations in large granular lymphocytic leukemia. *Blood* **2013**, *121*, 4541–4550, doi:10.1182/blood-2012-12-474577.
12. Küçük, C.; Jiang, B.; Hu, X.; Zhang, W.; Chan, J.K.C.; Xiao, W.; Lack, N.; Alkan, C.; Williams, J.C.; Avery, K.N.; et al. Activating mutations of STAT5B and STAT3 in lymphomas derived from γδ-T or NK cells. *Nat. Commun.* **2015**, *6*, 6025, doi:10.1038/ncomms7025.
13. de Araujo, E.D.; Erdogan, F.; Neubauer, H.A.; Meneksedag-Erol, D.; Manaswiyoungkul, P.; Eram, M.S.; Seo, H.S.; Qadree, A.K.; Israelian, J.; Orlova, A.; et al. Structural and functional consequences of the STAT5BN642H driver mutation. *Nat. Commun.* **2019**, *10*, 2517, doi:10.1038/s41467-019-10422-7.
14. Pham, H.T.T.; Maurer, B.; Prchal-Murphy, M.; Grausenburger, R.; Grundschober, E.; Javaheri, T.; Nivarthi, H.; Boersma, A.; Kolbe, T.; Elabd, M.; et al. STAT5BN642H is a driver mutation for T cell neoplasia. *J. Clin. Investig.* **2018**, *128*, 387–401, doi:10.1172/JCI94509.
15. Huang, C.-Y.; Lin, Y.-C.; Hsiao, W.-Y.; Liao, F.-H.; Huang, P.-Y.; Tan, T.-H. DUSP4 deficiency enhances CD25 expression and CD4^+^ T-cell proliferation without impeding T-cell development. *Eur. J. Immunol.* **2012**, *42*, 476–488, doi:10.1002/eji.201041295.
16. Hsiao, W.-Y.; Lin, Y.-C.; Liao, F.-H.; Chan, Y.-C.; Huang, C.-Y. Dual-specificity phosphatase 4 regulates STAT5 protein stability and helper T Cell polarization. *PLoS ONE* **2015**, *10*, e0145880, doi:10.1371/journal.pone.0145880.
17. Irie-Sasaki, J.; Sasaki, T.; Matsumoto, W.; Opavsky, A.; Cheng, M.; Welstead, G.; Griffiths, E.; Krawczyk, C.; Richardson, C.D.; Aitken, K.; et al. CD45 is a JAK phosphatase and negatively regulates cytokine receptor signalling. *Nature* **2001**, *409*, 349–354, doi:10.1038/35053086.
18. Simoncic, P.D.; Lee-Loy, A.; Barber, D.L.; Tremblay, M.L.; McGlade, C.J. The T cell protein tyrosine phosphatase is a negative regulator of janus family Kinases 1 and 3. *Curr. Biol.* **2002**, *12*, 446–453, doi:10.1016/S0960-9822(02)00697-8.
19. Xiao, W.; Hong, H.; Kawakami, Y.; Kato, Y.; Wu, D.; Yasudo, H.; Kimura, A.; Kubagawa, H.; Bertoli, L.F.; Davis, R.S.; et al. Tumor suppression by phospholipase C-beta3 via SHP-1-mediated dephosphorylation of Stat5. *Cancer Cell* **2009**, *16*, 161–171.
20. David, M.; Chen, H.E.; Goelz, S.; Larner, A.C.; Neel, B.G. Differential regulation of the alpha/beta interferon-stimulated Jak/Stat pathway by the SH2 domain-containing tyrosine phosphatase SHPTP1. *Mol. Cell. Biol.* **1995**, *15*, 7050–7058, doi:10.1128/mcb.15.12.7050.
21. Liau, N.P.D.; Laktyushin, A.; Lucet, I.S.; Murphy, J.M.; Yao, S.; Whitlock, E.; Callaghan, K.; Nicola, N.A.; Kershaw, N.J.; Babon, J.J. The molecular basis of JAK/STAT inhibition by SOCS1. *Nat. Commun.* **2018**, *9*, 1558, doi:10.1038/s41467-018-04013-1.
22. Demirel, Ö.; Balló, O.; Reddy, P.N.G.; Vakhrusheva, O.; Zhang, J.; Eichler, A.; Fernandes, R.; Badura, S.; Serve, H.; Brandts, C. SOCS1 function in BCR-ABL mediated myeloproliferative disease is dependent on the cytokine environment. *PLoS ONE* **2017**, *12*, e0180401, doi:10.1371/journal.pone.0180401.
23. Cacalano, N.A.; Sanden, D.; Johnston, J.A. Tyrosine-phosphorylated SOCS-3 inhibits STAT activation but binds to p120 RasGAP and activates Ras. *Nat. Cell Biol.* **2001**, *3*, 460–465, doi:10.1038/35074525.
24. Babon, J.J.; Kershaw, N.J.; Murphy, J.M.; Varghese, L.N.; Laktyushin, A.; Young, S.N.; Lucet, I.S.; Norton, R.S.; Nicola, N.A. Suppression of cytokine signaling by SOCS3: Characterization of the mode of inhibition and the basis of its specificity. *Immunity* **2012**, *36*, 239–250, doi:10.1016/j.immuni.2011.12.015.
25. Beier, U.H.; Wang, L.; Han, R.; Akimova, T.; Liu, Y.; Hancock, W.W. Histone deacetylases 6 and 9 and sirtuin-1 control Foxp^3+^ regulatory T cell function through shared and isoform-specific mechanisms. *Sci. Signal.* **2012**, *5*, ra45, doi:10.1126/scisignal.2002873.
26. Wickham, H.; François, R.; Henry, L.; Müller, K. Dplyr: A Grammar of Data Manipulation (version 0.8.1). Available online: https://cran.r-project.org/package=dplyr (accessed on 20 May 2019).
27. Wickham, H. *Ggplot2: Elegant Graphics for Data Analysis*; Springer: New York, NY, USA, 2016.
28. R Core Team. *R: A Language and Environment for Statistical Computing*; The R Foundation for Statistical Computing: Vienna, Austria, 2018.
29. Wickham, H.; Bryan, J. Readxl: Read Excel Files (version 1.3.1). Available online: https://cran.r-project.org/package=readxl (accessed on 22 May 2019).
30. Therneau, T.M.; Grambsch, P.M. *Modeling Survival Data: Extending the Cox Model*; Springer: New York, NY, USA, 2000.
31. Kassambara, A.; Kosinski, M.; Biecek, P. Survminer: Drawing Survival Curves using “ggplot2” (version 0.4.6). Available online: https://cran.r-project.org/package=survminer (accessed on 5 September 2019).
32. Wickham, H.; Henry, L.; Luciani, T.J.; Decorde, M.; Lise, V. *Svglite: An “SVG” Graphics Device* (version 1.2.2). Available online: https://cran.r-project.org/package=svglite (accessed on 22 May 2019).
33. Gehlenborg, N. UpSetR: A More Scalable Alternative to Venn and Euler Diagrams for Visualizing Intersecting Sets (version 1.4.0). Available online: https://cran.r-project.org/package=UpSetR (accessed on 7 September.2019).
34. Dragulescu, A.A.; Arendt, C. XLSX: Read, Write, Format Excel 2007 and Excel 97/2000/XP/2003 Files (version 0.6.1). Available online: https://cran.r-project.org/package=xlsx (accessed on 22 May 2019).
35. Durinck, S.; Moreau, Y.; Kasprzyk, A.; Davis, S.; De Moor, B.; Brazma, A.; Huber, W. BioMart and Bioconductor: A powerful link between biological databases and microarray data analysis. *Bioinformatics* **2005**, *21*, 3439–3440, doi:10.1093/bioinformatics/bti525.
36. Lawrence, M.; Huber, W.; Pagès, H.; Aboyoun, P.; Carlson, M.; Gentleman, R.; Morgan, M.T.; Carey, V.J. Software for computing and annotating genomic ranges. *PLoS Comput. Biol.* **2013**, *9*, e1003118, doi:10.1371/journal.pcbi.1003118.
37. Bolger, A.M.; Lohse, M.; Usadel, B. Trimmomatic: A flexible trimmer for Illumina sequence data. *Bioinformatics* **2014**, *30*, 2114–2120, doi:10.1093/bioinformatics/btu170.
38. MacDonald, J.R.; Ziman, R.; Yuen, R.K.C.; Feuk, L.; Scherer, S.W. The database of genomic variants: A curated collection of structural variation in the human genome. *Nucleic Acids Res.* **2014**, *42*, D986–D992, doi:10.1093/nar/gkt958.
39. Li, H.; Durbin, R. Fast and accurate short read alignment with Burrows-Wheeler transform. *Bioinformatics* **2009**, *25*, 1754–1760, doi:10.1093/bioinformatics/btp324.
40. DePristo, M.A.; Banks, E.; Poplin, R.; Garimella, K. V.; Maguire, J.R.; Hartl, C.; Philippakis, A.A.; del Angel, G.; Rivas, M.A.; Hanna, M.; et al. A framework for variation discovery and genotyping using next-generation DNA sequencing data. *Nat. Genet.* **2011**, *43*, 491–498, doi:10.1038/ng.806.
41. Li, H.; Handsaker, B.; Wysoker, A.; Fennell, T.; Ruan, J.; Homer, N.; Marth, G.; Abecasis, G.; Durbin, R.; 1000 Genome Project Data Processing Subgroup. The Sequence Alignment/Map format and SAMtools. *Bioinformatics* **2009**, *25*, 2078–2079, doi:10.1093/bioinformatics/btp352.
42. McKenna, A.; Hanna, M.; Banks, E.; Sivachenko, A.; Cibulskis, K.; Kernytsky, A.; Garimella, K.; Altshuler, D.; Gabriel, S.; Daly, M.; et al. The genome analysis toolkit: A MapReduce framework for analyzing next-generation DNA sequencing data. *Genome Res.* **2010**, *20*, 1297–1303, doi:10.1101/gr.107524.110.
43. Koboldt, D.C.; Zhang, Q.; Larson, D.E.; Shen, D.; McLellan, M.D.; Lin, L.; Miller, C.A.; Mardis, E.R.; Ding, L.; Wilson, R.K. VarScan 2: Somatic mutation and copy number alteration discovery in cancer by exome sequencing. *Genome Res.* **2012**, *22*, 568–576, doi:10.1101/gr.129684.111.
44. Cingolani, P.; Platts, A.; Wang, L.L.; Coon, M.; Nguyen, T.; Wang, L.; Land, S.J.; Lu, X.; Ruden, D.M. A program for annotating and predicting the effects of single nucleotide polymorphisms, SnpEff. *Fly (Austin)* **2012**, *6*, 80–92, doi:10.4161/fly.19695.
45. 1000 Genomes Project Consortium; Abecasis, G.R.; Auton, A.; Brooks, L.D.; DePristo, M.A.; Durbin, R.M.; Handsaker, R.E.; Kang, H.M.; Marth, G.T.; McVean, G.A. An integrated map of genetic variation from 1,092 human genomes. *Nature* **2012**, *491*, 56–65, doi:10.1038/nature11632.
46. Sherry, S.T.; Ward, M.-H.; Kholodov, M.; Baker, J.; Phan, L.; Smigielski, E.M.; Sirotkin, K. dbSNP: The NCBI database of genetic variation. *Nucleic Acids Res.* **2001**, *29*, 308–311, doi:10.1093/NAR/29.1.308.
47. Cibulskis, K.; Lawrence, M.S.; Carter, S.L.; Sivachenko, A.; Jaffe, D.; Sougnez, C.; Gabriel, S.; Meyerson, M.; Lander, E.S.; Getz, G. Sensitive detection of somatic point mutations in impure and heterogeneous cancer samples. *Nat. Biotechnol.* **2013**, *31*, 213–219, doi:10.1038/nbt.2514.
48. Korn, J.M.; Kuruvilla, F.G.; McCarroll, S.A.; Wysoker, A.; Nemesh, J.; Cawley, S.; Hubbell, E.; Veitch, J.; Collins, P.J.; Darvishi, K.; et al. Integrated genotype calling and association analysis of SNPs, common copy number polymorphisms and rare CNVs. *Nat. Genet.* **2008**, *40*, 1253–1260, doi:10.1038/ng.237.
49. Min, H.; Zhou, F.; Jui, S.; Wang, T.; Chen, X. Affymetrix^®^ canary algorithm version 1.0. *Affymetrix White Pap.* **2004**, 1–23.
50. Thorvaldsdottir, H.; Robinson, J.T.; Mesirov, J.P. Integrative Genomics Viewer (IGV): High-performance genomics data visualization and exploration. *Brief. Bioinform.* **2013**, *14*, 178–192, doi:10.1093/bib/bbs017.
51. Hunt, S.E.; McLaren, W.; Gil, L.; Thormann, A.; Schuilenburg, H.; Sheppard, D.; Parton, A.; Armean, I.M.; Trevanion, S.J.; Flicek, P.; et al. Ensembl variation resources. *Database* **2018**, doi:10.1093/database/bay119.

| 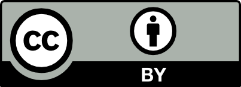 | © 2019 by the authors. Licensee MDPI, Basel, Switzerland. This article is an open access article distributed under the terms and conditions of the Creative Commons Attribution (CC BY) license (http://creativecommons.org/licenses/by/4.0/). |
| --- | --- |
